# 6-Gingerol Alleviates Neonatal Hypoxic-Ischemic Cerebral and White Matter Injury and Contributes to Functional Recovery

**DOI:** 10.3389/fphar.2021.707772

**Published:** 2021-09-22

**Authors:** Man Zhao, Yuan Yao, Jingyi Du, Liang Kong, Tiantian Zhao, Dong Wu, Lajie Man, Wenjuan Zhou

**Affiliations:** ^1^ Key Laboratory for Experimental Teratology of Ministry of Education, Shandong Key Laboratory of Mental Disorders, Key Laboratory of Birth Regulation and Control Technology of National Health Commission of China, Department of Anatomy and Histoembryology, School of Basic Medical Sciences, Cheeloo College of Medicine, Shandong University, Jinan, China; ^2^ Centre for Sports and Exercise Science, School of Sport, Rehabilitation and Exercise Sciences, University of Essex, Colchester, United Kingdom; ^3^ Department of Clinical Laboratory, Affiliated Hospital of Shandong University of Traditional Chinese Medicine, Jinan, China

**Keywords:** hypoxic-ischemic brain injury, neuroinflammation, motor deficits, oligodendrocytes maturation, H3K27me3

## Abstract

Hypoxic-ischemic encephalopathy (HIE) is one main cause of neonatal death and disability, causing substantial injury to white and gray matter, which can lead to severe neurobehavioral dysfunction, including intellectual disability and dyskinesia. Inflammation, nerve cell death, and white matter injury are important factors in the pathological process of HIE. 6-Gingerol is a ginger extract, which reduces inflammatory response and cell death. However, the role of 6-Gingerol in neonatal hypoxic-ischemic brain injury (HIBI) remains unknown. In this study, we constructed a mouse HIBI model and analyzed the protective effect of 6-Gingerol on HIBI by using behavioral tests, histological staining, qPCR and western blot. Here, we found that 6-Gingerol treatment could alleviate HIBI and improve short-term reflex performance, which is closely related to cell death and neuroinflammation. Additionally, 6-Gingerol reduced neuronal apoptosis, pro-inflammatory factor release, as well as microglial activation. Furthermore, 6-Gingerol significantly improved motor disability, which is associated with white matter damage. Thus, our results showed that 6-Gingerol could reduce the loss of myelin sheaths, alleviate cell death of oligodendrocytes, and stimulate the maturation of oligodendrocytes. In terms of mechanism, we found that 6-Gingerol decreased histone H3K27me3 levels, activated AKT pathway and inhibited the activation of ERK and NF-κB pathway at 3 days post-HIBI. Taken together, our data clearly indicate that 6-Gingerol plays a neuroprotective role against HIBI by epigenetic modification and regulation of AKT, ERK, and NF-κB pathways, inhibiting inflammatory responses and reducing cell death.

## Introduction

Hypoxic-ischemic encephalopathy (HIE) refers to neonatal brain damage caused by perinatal asphyxia. It is a major cause of death and disability in infants globally. The incidence of HIE is 1–8 per 1,000 live births in developed countries and up to 26 per 1,000 in developing countries (Kurinczuk et al., 2010). Infants with HIE usually develop long-term neurological sequelae, including motor deficits, gastrointestinal and nutritional problems, orthopaedic problems, sensory and cognitive impairments, and so on, which affect the health of infants and impose a heavy burden on society and the families of these infants (Eunson, 2015). Application of mild hypothermia therapy is advised for moderate or severe HIE within the first 6 h post-HIE. At present, mild hypothermia treatment of HIE is effective to some extent, but the therapeutic time window is too narrow to allow a timely start. (Gluckman et al., 2005; Graham et al., 2008; Edwards et al., 2010). Therefore, there is an urgent need to explore new effective therapies to minimize brain injury and neurological sequelae of HIE in neonates.

Hypoxic-ischemic brain injury (HIBI) usually leads to HIE. Many studies have reported that the pathogenesis of HIBI is complex, and includes various forms of nerve cell death, such as necrosis, apoptosis, autophagy, excitatory toxicity, oxidative stress, and neuroinflammation (Wen et al., 2008; Knox et al., 2013; Douglas-Escobar and Weiss, 2015; Rainaldi and Perlman, 2016; Arteaga et al., 2017; Li et al., 2017). Neuronal apoptosis is a major pathological feature in HIBI, which is activated in the early stage and continues for several weeks after HIBI(Douglas-Escobar and Weiss, 2015). A number of studies have highlighted the inhibition of apoptosis as an effective treatment for HIBI(Carlsson et al., 2011; Rodriguez et al., 2018; Li et al., 2020). Moreover, inflammation has been deemed to be a pivotal contributor to the pathophysiology of HIBI. Brain resident cells are activated immediately following HIBI, after which the injured parenchyma is infiltrated by circulating peripheral leukocytes. As a type of resident innate immune cell in the central nervous system, microglial cells are activated after HIBI and secrete cytokines that lead to secondary neuronal damage (Li et al., 2017). Therefore, reducing neuroinflammation is a promising therapeutic target for HIBI. In addition, much attention has been paid to white matter, which is susceptible to hypoxic-ischemic injury, and can result in demyelination (Drobyshevsky et al., 2007; Epelman et al., 2012). White matter injury in the newborn brain can lead to cerebral palsy, motor and cognitive disability (Fancy et al., 2011). Accordingly, the recovery of white matter injury following HIBI, is a promising therapeutic candidate for improving HIBI outcomes.

Currently, use of traditional Chinese medicine for the treatment of nervous system diseases has increasingly drawn attention. Ginger, a popular food and herbal medicine, has a history of more than 2000 years in China, which has been used as treatment for many diseases, such as tumors, muscle diseases, and cardiovascular diseases, with marked positive effects (Semwal et al., 2015; Montserrat-de la Paz et al., 2018; Borcan et al., 2019; Mahomoodally et al., 2021). 6-Gingerol, the major pungent component of fresh ginger, is a phenolic substance with a variety of biological properties: it exerts anti-inflammatory, antioxidant, anti-tumor, antibiofilm, and antivirus activities (Lee et al., 2018; Han et al., 2020; Xu et al., 2020). 6-Gingerol is able to penetrate the blood‒brain barrier, which indicates that it can have an effect on the central nervous system (Simon et al., 2020). A recent study has shown that 6-Gingerol can inhibit the increase in GFAP and TNF-α levels and can suppress lipopolysaccharide (LPS)-induced astrocyte overactivation in rat brain, and thereby alleviate neuroinflammation and cognitive impairment (Zhang et al., 2018). Moreover, 6-Gingerol can regulate the activation of dendritic cells through NF-κB and MAPK signaling pathways, to inhibit the infiltration of inflammatory cells into the central nervous system of animals with experimental autoimmune encephalomyelitis (Han et al., 2019). However, the effect of 6-Gingerol on neonatal HIBI remains unclear. In the present study, the main purpose was to determine whether 6-Gingerol can alleviate brain damage and neurological deficits in a mouse model of neonatal HIBI and to explore its mechanism.

## Materials and Methods

### HIBI Model and Drugs Administration

All animal care and experiments were approved by the National Institutes of Health Guide for the Care and Use of Laboratory Animals and the Institutional Animal Care and Use Committees of Shandong University. The female Kunming mice (50–60 g), with their 10–12 pups were purchased from Shandong University Laboratory Animal Center.

The HIBI model in this study was the Rice‒Vannucci Model with minor changes (Rice et al., 1981). In brief, 7-day-old mouse pups were anesthetized with 2–3% isoflurane (RWD, Shenzhen, China). The neck skin was disinfected, and a longitudinal incision was made in the middle of the neck with a scalpel. After the neck tissue was removed, the right common carotid artery was exposed and ligated with No. 4-0 surgical sutures. After surgery, the mouse pups were returned to the dam for 1 h, and then placed in a 37°C hypoxic incubator (HERA Cell240; Thermo Fisher Scientific, Waltham, MA, United States ) under 8% oxygen, and 92% nitrogen for 90 min (Xin et al., 2018; Wang et al., 2021). In Sham group, the right common carotid artery was exposed without carotid artery ligation or hypoxia treatment.

6-Gingerol (DESITE, Chengdu, China) was dissolved in sterile normal saline with 1% dimethyl sulfoxide (Solarbio, Beijing, China). Most previous studies have shown that drug pre-treatment offer better protection against brain injury induced by HIBI. At the same time, 3 days after HIBI is not only the acute phase of brain injury, but also the key period for treatment (Feng et al., 2014; Xin et al., 2018; Zhang et al., 2018; Gravina et al., 2020; Wang et al., 2021). Therefore, 6-Gingerol was administered daily via intraperitoneal injection 24 h prior to HIBI, until 3 days post-HIBI injury. For short-term morphological and biochemical assessment, animal euthanasia and perfusion were performed 12 h after the last treatment. In our preliminary experiment, we found that the 6-Gingerol had no effect on brain structure in Sham group. According to previous studies, animals were divided into three groups: Sham group, HIBI group and HIBI+6G group (Zhang et al., 2018). The HIBI+6G group received different doses of 6-Gingerol (1 mg/kg, 2 mg/kg, 4 mg/kg) to determine the best therapeutic dose. Of these doses, 2 mg/kg 6-Gingerol appeared to optimize the minimum effective concentration. Therefore, except for the drug concentration screening experiment, a dose of 2 mg/kg was used to investigate the effect of 6-Gingerol on HIBI outcomes in our study. The Sham group and HIBI group were injected with same volume of sterile normal saline according to the pups’ weight. Animals were divided into two big subgroups: some animals were euthanized earlier for short-term morphological and biochemical assessment at 3 days after HIBI insult (P10); some animals were raised to adolescence for long-term behavioral and morphological assessment at P35.

### Brain Infarct Area Measurement and Brain Water Content Detection

Brain infarct area was measured by triphenyl tetrazolium chloride (TTC) staining 3 days after HIBI. Briefly, 2-mm-thick coronal slices of brain were immersed in 2% TTC solution (Solarbio, Beijing, China), in a dark place, at 37°C, for 30 min. Then, the infarction area was analyzed by using ImageJ software (National Institutes of Health, Bethesda, MD, United States ). *n* = 3–4 per group.

Brains were selected and weighed immediately at 3-day post HIBI. Then, the brains were placed in an oven (100°C) for 48 h and weighed again. The brain water content was calculated by the following formula: Brain water content (%) = [(wet weight–dry weight)/wet weight] × 100%.

### Histological Staining

The pups were euthanized and perfused with normal saline, followed by 4% paraformaldehyde (PFA) at 3 days after HIBI. The pups’ brains were removed and infiltrated with 4% PFA for 24 h. Using xylene and ethanol for gradient dehydration, the brains were embedded in paraffin and 4-μm-thick coronal sections were prepared using a microtome (LEICA RM 2235, Leica Biosystems, Wetzlar, Germany). Hematoxylin‒eosin (HE) and Nissl staining (KeyGEN BioTECH, Jiangsu, China) were performed, according to manufacturer’s instructions, to observe histopathological changes.

Briefly, after using xylene as dewaxing medium and an ethanol gradient as a hydrating agent, brain slices were permeabilized with 0.5% Triton X-100, blocked in 10% donkey serum, and incubated with primary antibodies: goat anti-Olig2 (1:20, R and D Systems, Minneapolis, MN, United States ), rabbit anti-PDGFR-α (1:600, Cell Signaling Technologies; Danvers, MA, United States ), rabbit anti-APC (1:50, Beyotime Biotechnology; Shanghai, China), rabbit anti-active caspase3 (1:200, Abcam, Cambridge, MA, United States ), goat anti-Iba-1 (1:200, Abcam, Cambridge, MA, United States ), rabbit anti-TNF-α (1:200, Bioss, Beijing, China), rabbit anti-IL-1β (1:200, Affinity Biosciences, Cincinnati, OH, United States ) and rabbit anti-myelin basic protein (MBP; 1:600, Cell Signaling Technologies; Danvers, MA, United States ). Then, the sections were incubated with secondary antibodies, including donkey anti-goat IFKine Green and donkey anti-rabbit IFKine Red. Next, they were stained with 4′, 6-diamidino-2-phenylindole (DAPI). Apoptosis Detection Kit (KeyGEN BioTECH, Nanjing, China) was used for TUNEL staining. Representative images were captured using the upright fluorescence microscope (Olympus BX51; Olympus, Shinjuku, Japan). *n* = 6 per group.

Luxol Fast Blue (LFB) staining was performed to evaluate demyelination of the white matter at 4-week post-HIBI. Briefly, paraffin sections were prepared as previously described. After dewaxing and hydrating, brain sections were immersed in LFB solution at 65°C for 4 h in a dark place and were then treated with lithium carbonate solution for 10 s and dehydrated with ethanol. Photos were captured under an optical microscope (Olympus BX51; Olympus, Shinjuku, Japan).

### Western Blotting

Brain samples were selected and homogenized in RIPA buffer containing protease and phosphatase inhibitors for 30 min on ice. The supernatants were collected by centrifugation at 13,000 × revolutions per minute (rpm) at 4°C. Next, protein concentration was detected by using bicinchoninic acid (BCA) assay (Pierce Biotechnology, Waltham, MA, United States ). The loading buffer was added to the samples, which were then boiled at 98°C for 5 min. Samples containing an equal amount of protein were separated by SDS-PAGE. After protein separation, the protein of interest were transferred to polyvinylidene fluoride (PVDF) membranes and were incubated with the following primary antibodies: mouse anti-Bcl-2 (1:500, Santa Cruz Biotechnology, Inc., Dallas, Texas, United States ), rabbit anti-Bax (1:1,000, Cell Signaling Technologies), rabbit anti-Cleaved PARP I (1:1,000, Abcam, Cambridge, MA, United States ), rabbit anti-IL-1β (1:500, Affinity Biosciences, Cincinnati, OH, United States ), rabbit anti-TNF-α (1:500, Bioss, Beijing, China), rabbit anti-MBP (1:1,000, Cell Signaling Technologies), rabbit anti-acetyl-histone (1:500, Cell Signaling Technologies), rabbit anti-histone H3 (1:1,000, Cell Signaling Technologies), rabbit anti-tri-metryl-histone H3 (K27) (1:1,000, Cell Signaling Technologies), rabbit anti-tri-metryl-histone H3 (K9) (1:1,000, Cell Signaling Technologies), rabbit anti-tri-metryl-histone H3 (K4) (1:1,000, Cell Signaling Technologies), rabbit anti-AKT (1:1,000, Cell Signaling Technologies), rabbit anti-phosphorylated-AKT (1:1,000, Cell Signaling Technologies), rabbit anti-ERK1/2 (1:1,000, Beyotime Biotechnology; Shanghai, China), rabbit anti-phosphorylated-ERK1/2 (1:1,000, Beyotime Biotechnology; Shanghai, China), mouse anti-NF-κB (1:1,000, Cell Signaling Technologies), mouse anti-phosphorylated-NF-κB (1:1,000, Cell Signaling Technologies), mouse anti-β-actin (1:2000, TransGen Biotech, Beijing, China). *n* = 3-5 per group.

### RNA Extraction and Quantitative Real-Time Polymerase Chain Reaction

Brain samples were obtained and homogenized with TRIZOL solution (Invitrogen, Waltham, MA, United States ). Then, total RNA was extracted by using *TransZol*™ Up Plus RNA Kit (TransGen Biotech, Beijing, China) according to the manufacturer’s protocol. The concentration of RNA was determined by spectrophotometer (NanoDrop 2000; Thermo Fisher Scientific) and SYBR Green Realtime PCR Master Mix (TOYOBO, Osaka, Japan) was used for quantitative real-time PCR. The expression of target genes was analyzed by 2^−ΔΔCT^ method and β-actin was used as a reference gene. *n* = 3–4 per group. The primer sequences were listed in Table 1.

**TABLE 1:** PCR primers sequence.

Gene	Forward	Reverse
IL-1β	AGCATCCAGCTTCAAATC	CTTCTCCACAGCCACAAT
TNF-α	TCT​CAT​TCC​TGC​TTG​TGG​C	CAC​TTG​GTG​GTT​TGC​TAC​G
β-actin	CGT​TGA​CAT​CCG​TAA​AGA​CCT​C	CCA​CCG​ATC​CAC​ACA​GAG​TAC

### Neurobehavioral Assessment

According to previous studies, neurological reflexes, including negative geotaxis, righting reflex, and hind-limb suspension, were tested 3 days after HIBI (P10), *n* = 8 per group. These reflect short-term neurobehaviors (Xin et al., 2018). Mice were subjected to a rotarod test and open-field test at 4 week after HIBI (P35, i.e., adolescence), which is a key time-point for behavioral detection, to reflect long-term neurobehavior (Xin et al., 2018; Ke et al., 2020), *n* = 5-6 per group. Brain weight and atrophy were also examined at 4 weeks post-HIBI, n = 6 per group. A schematic diagram of the experimental procedure is as follows. .

#### Negative Geotaxis

At 3-days post-HIBI, pups were placed on a 25-cm-long tilt board (45°), with a head-down position. The time required for pups to turn 180°was recorded during the 60-s test. The test was repeated three times for each pup and the average time of three tests was used as the final result, to avoid randomness (Ke et al., 2020; Wang et al., 2021).

#### Righting Reflex

At 3-days post-HIBI, pups were placed in a supine position and the time taken to flip to the prone position was recorded. Test was repeated three times for each pup and the average time of three tests was used as the final result (Ke et al., 2020; Wang et al., 2021).

#### Hind-Limb Suspension

At 3-days post-HIBI, pups were suspended in a hollow tube placed vertically by their hind limbs. The suspension time was recorded. Test was repeated three times for each pup and the average time of three tests was used as the final result (Ke et al., 2020; Wang et al., 2021).

#### Rotarod Test

In order to evaluate the motor coordination of mice, rotarod test (RT) was used at 4 weeks after HIBI, as previously reported, which is a key time-point for behavioral detection (Ke et al., 2020). Briefly, 1 day before the formal experiment, each mouse was trained at a fixed rate of 5 rpm for 5 min (Mohebbi et al., 2020). On testing day, each mouse was trained at a fixed rate of 10 rpm for 10s, and the rotation speed was gradually accelerated from 10 to 40 rpm for 120 s. Before the formal experiment, each mouse was trained at a fixed rate of 10 rpm for 10 s. After training, the rotation speed was gradually accelerated from 10 to 40 rpm for 120 s, and the time on the rod was recorded by using a rotating rod fatigue instrument (Sansbio, Jiangsu, China). The duration of the test was 120 s. Test was repeated three times for each pup and the average time of three tests was used as the final result (Xin et al., 2018; Wang et al., 2021).

#### Open-Field Test

Open-field test (OFT) was performed at 4 weeks after HIBI to assess the voluntary motor abilities of mice in certain areas. OFT equipment consisted of a blue plastic box without a lid (diameter, 40 cm; depth, 40 cm). Each mouse was placed in the center of the box and allowed to roam free for 10 min. A video analysis system (EthoVision XT5) was used to record the speed and distance of mice moving in the area.

### Statistical Analysis

Statistical analysis was performed using GraphPad Prism 8.0 software. Values were expressed as mean ± SEM. In this study, only 6-Gingerol treatment was considered as the study factor. Therefore, data statistical significance was calculated by one-way ANOVA, followed by Tukey’s test. *p* < 0.05 was considered as statistically significant.

## Results

### 6-Gingerol Reduces Brain Damage and Improves Short-Term Neurological Function

**FIGURE 1:**
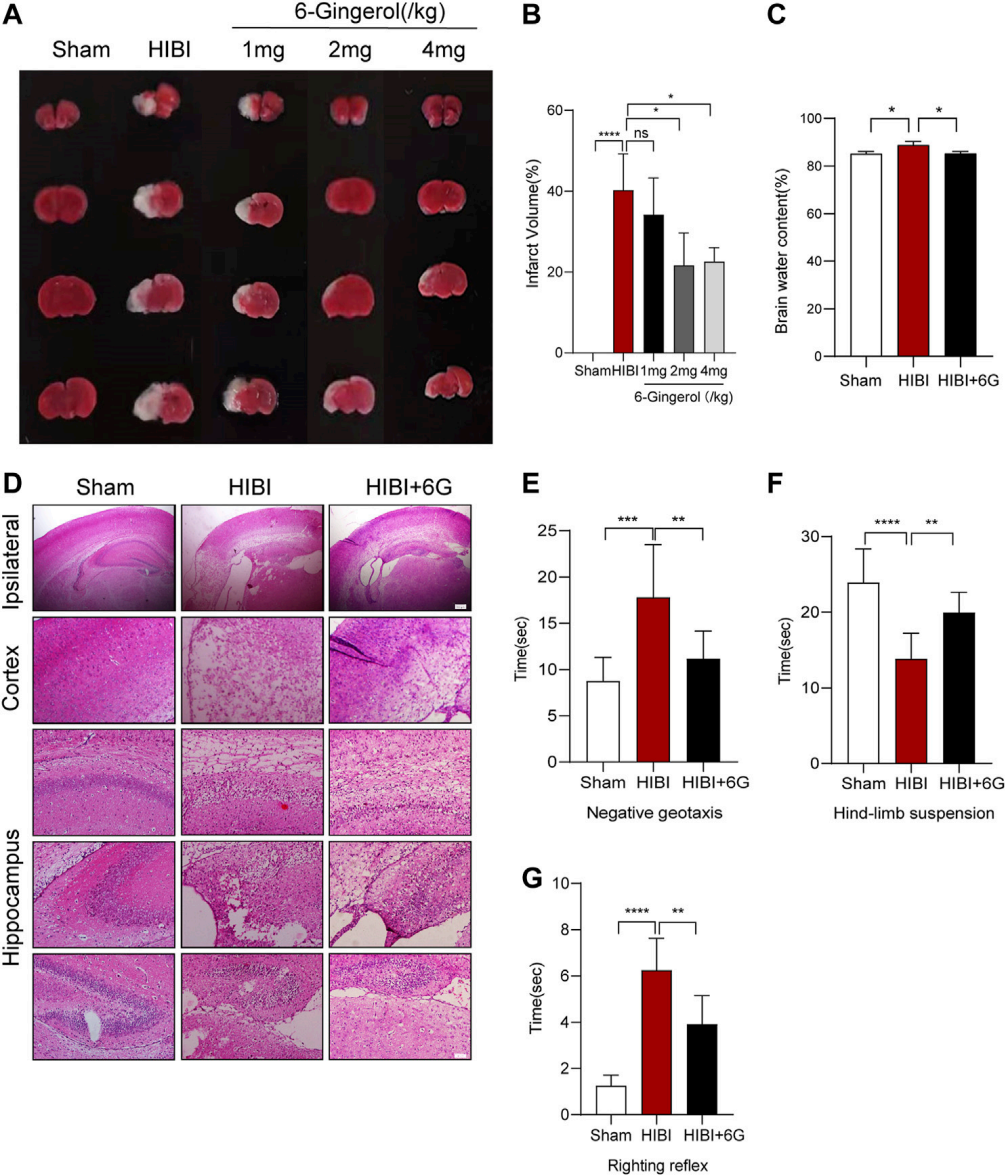
Effect of 6-Gingerol on brain infarct area and neurological reflexes. **(A)** Triphenyl tetrazolium chloride (TTC) staining of the Sham, HIBI and HIBI+6G group with different 6-Gingerol doses for treatment. **(B)** Calculation of brain infarct area shown by TTC staining from Figure 1A. **(C)** Calculation of brain water content. **(D)** Representative pictures of hematoxylin and eosin (HE) staining. *n* = 3-4 per group in A-D. lpsilateral, scale bar = 200 μm. Cortex and hippocampus, scale bar = 50 μm. **(E–G)** Performance of negative geotaxis, hind-limb suspension and righting reflex. *n* = 8 per group. Data represent the mean ± SEM. *p < 0.05, **p < 0.01, ***p < 0.001, ****p < 0.0001.

To investigate whether 6-Gingerol could alleviate HIBI, 1 mg/kg, 2 mg/kg and 4 mg/kg 6-Gingerol were administered once per day for 4 days via intraperitoneal injection after HIBI. TTC staining was performed to quantify the cerebral infarction volume in different groups, where normal brain tissue stains red and the ischemic area remains white. As shown in Figures 1A,B, mice in the Sham group had no obvious cerebral ischemic area, while HIBI substantially increased the cerebral ischemic area (*p* < 0.0001). In the HIBI+6G groups, 1 mg/kg 6-Gingerol had no effect on brain damage, while the application of 2 mg/kg and 4 mg/kg 6-Gingerol could significantly alleviate the percentage of the cerebral ischemic area induced by HIBI (*p* = 0.016, *p* = 0.036, respectively). Of these doses, 2 mg/kg 6-Gingerol appeared to optimize the minimum effective concentration. Therefore, except for the drug concentration screening experiment, a dose of 2 mg/kg was used to investigate the effect of 6-Gingerol on HIBI outcomes in our study.

Then, we further assessed cerebral edema by detecting brain water content. Brain water in HIBI group was increased by 4.266% compared with Sham group (*p* = 0.019), while after 6-Gingerol treatment, the brain water content of mice was decreased at 3-day post-HIBI, only 0.078% higher than that in Sham group (Figure 1C, *p* = 0.020). HE staining was used to assess histomorphological injury. Neural cells in Sham group were stained clearly with regular cell shape. However, the arrangement of neural cells became loose and disordered after HIBI, which was alleviated by 6-Gingerol treatment (Figure 1D). Next, the effect of 6-Gingerol treatment on short-term neurobehavioral function post-HIBI was evaluated at P10. In Figure 1E, the negative geotaxis time in the HIBI group significantly increased compared with the Sham group (*p* = 0.001). Treatment with 6-Gingerol could significantly reduce the negative geotaxis response time (*p* = 0.009). On the hind-limb suspension test, the HIBI group showed a slightly shorter duration of suspension than the Sham group (*p* < 0.0001), while 6-Gingerol treatment significantly improved the suspension time as compared with the HIBI group (*p* = 0.007) (Figure 1F). As shown in Figure 1G, the HIBI group took longer to complete the righting reflex than the Sham group (*p* < 0.0001). In contrast, 6-Gingerol treatment significantly decreased the righting reflex time in the HIBI group (*p* = 0.001).

### 6-Gingerol Alleviates HIBI-Induced Cell Apoptosis

**FIGURE 2:**
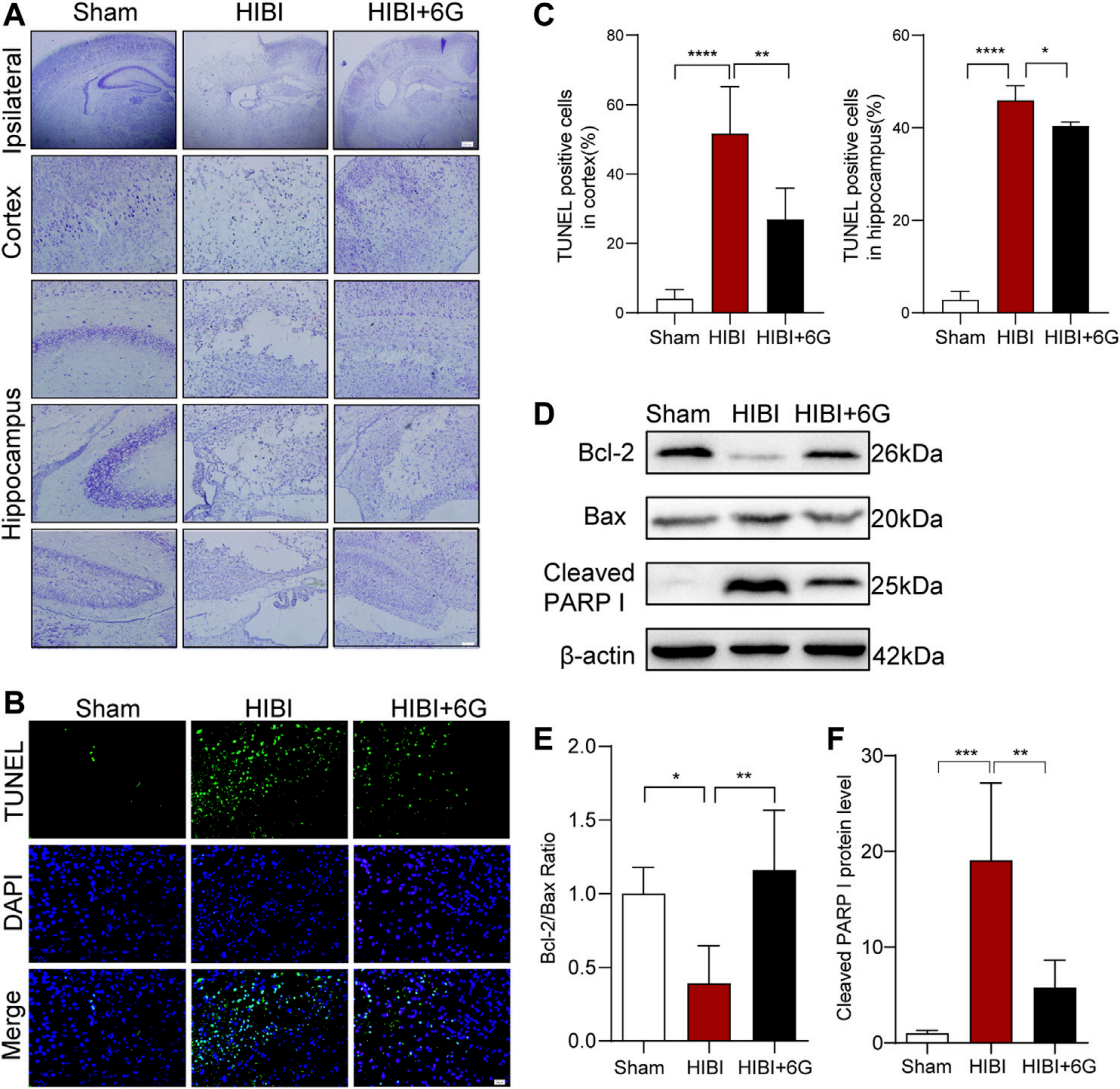
6-Gingerol alleviates HIBI-induced neuronal apoptosis. **(A)** Nissl staining of the cortex and hippocampus in each group at 3-day post-HIBI. lpsilateral, scale bar = 200 μm. Cortex and hippocampus, scale bar = 50 μm. **(B)** Representative TUNEL-stained (green) and DAPI-stained (blue) brain sections of the cortex 3-day post-HIBI. Scale bar = 20 μm. **(C)** Quantitative analysis of the percentage of TUNEL-positive cells in the cortex and hippocampus. *n* = 6 per group in A-C. **(D–F)** Representative western blot images and quantification of Bcl-2, Bax and Cleaved PARP I expressions at 3-day post- HIBI. *n* = 5 per group. Data represent the mean ± SEM. *p < 0.05, **p < 0.01, ***p < 0.001, ****p < 0.0001.

Nissl and TUNEL staining were carried out at 3-day post-HIBI to test the effects of 6-Gingerol treatment on cell apoptosis after HIBI. As shown in Figure 2A, in the Sham group, the neurons of the cortex and hippocampus presented a clear borderline and were arranged neatly. Nissl bodies were large and densely-distributed. HIBI resulted in brain tissue loss within the ipsilateral side. The remaining neurons were arranged disorderly with pyknotic nuclei and disappearance of Nissl’s bodies was also observed. However, compared with HIBI group, the extent of neuronal necrosis in the HIBI+6G group was alleviated, and the number of neurons and Nissl bodies was increased. In Figures 2B,C, more TUNEL-positive cells were seen in the HIBI group than in that of the Sham group (*p* < 0.0001). After 6-Gingerol treatment, the number of apoptotic cells was reduced to a large extent (*p* = 0.001, *p* = 0.048, respectively). To further confirm the anti-apoptotic effects of 6-Gingerol against HIBI, the expression levels of Bcl-2, Bax and Cleaved PARP I in the brain were examined by western blot (P10). As shown in Figures 2D–F, compared with that in the Sham group, the ratio of Bcl-2/Bax in the HIBI group was decreased (*p* = 0.018), and the expression level of Cleaved PARP I was increased (*p* < 0.001). 6-Gingerol treatment markedly reversed the effects of the HIBI (*p* = 0.004, *p* = 0.003, respectively), suggesting that 6-Gingerol played an important anti-apoptotic role.

### 6-Gingerol Inhibits Pro-inflammatory Gene Expression and Microglial Activation

**FIGURE 3:**
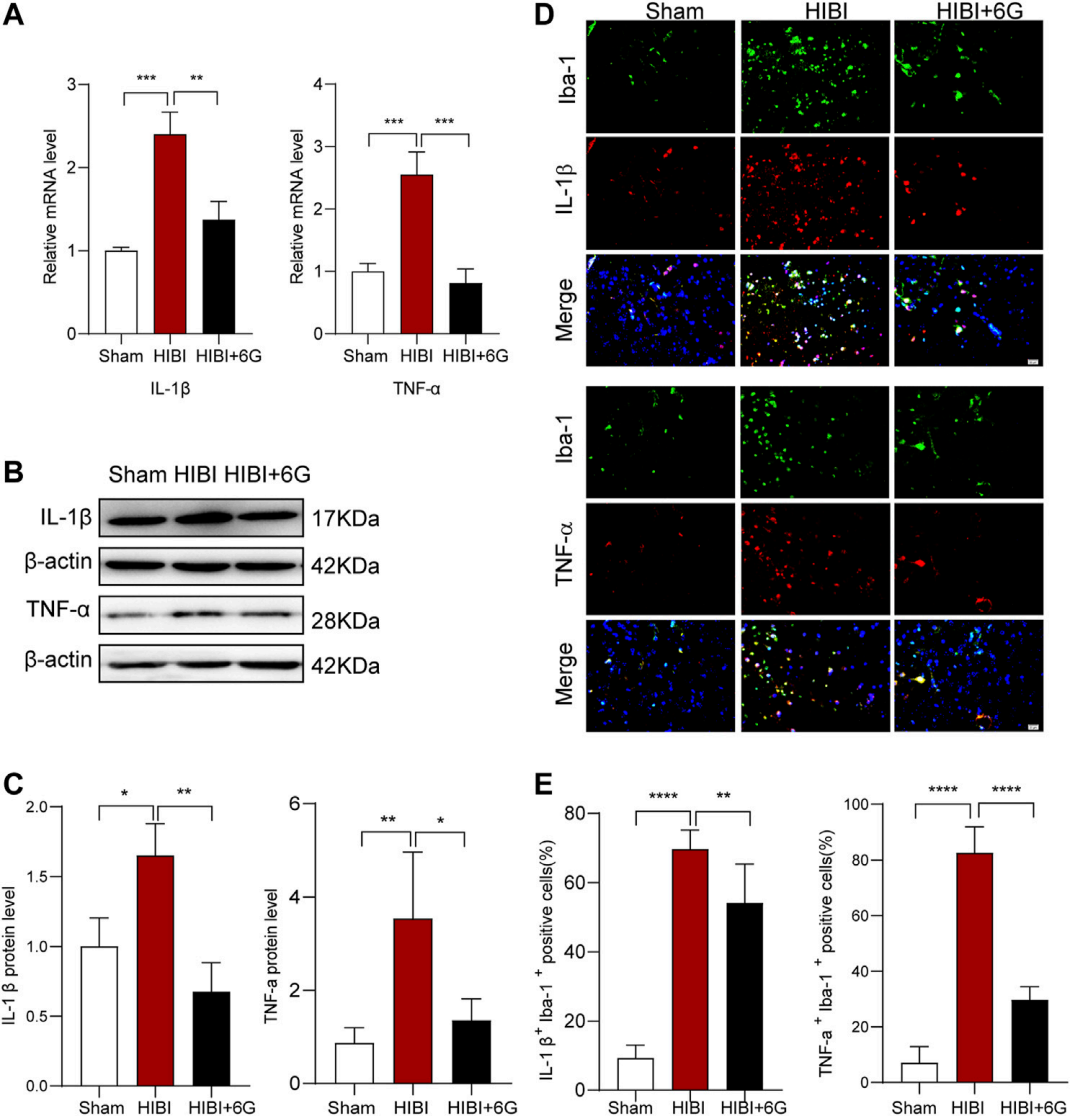
6-Gingerol inhibits pro-inflammatory gene expression and microglial activation post-HIBI. **(A)** Quantification of the mRNA level of IL-1β and TNF-α in the brain at 3-day post-HIBI. *n* = 3 per group. **(B–C)** Representative western blot images and quantification of IL-1β and TNF-α expression at 3-day post-HIBI. *n* = 3–4 per group. **(D–E)** Immunofluorescent staining of IL-1β Iba-1 and TNF-α Iba-1, and quantification of IL-1α+ Iba-1+ and TNF-α+ Iba-1+ cells at 3-day post-HIBI, *n* = 6 per group. Scale bar = 20 μm. Data represent the mean ± SEM. *p < 0.05, **p < 0.01, ***p < 0.001, ****p < 0.0001.

In order to evaluate whether 6-Gingerol treatment could regulate inflammatory reaction, we measured the mRNA expression of pro-inflammatory cytokines at 3-days post-HIBI (P10). As shown in Figure 3A, the mRNA levels of IL-1β and TNF-α were found to be strongly upregulated in the HIBI group (*p* < 0.001), while 6-Gingerol treatment reduced the expression levels of IL-1β and TNF-α post-HIBI (*p* = 0.002, *p* < 0.001, respectively). Meanwhile, the protein levels of IL-1β and TNF-α post-HIBI were also decreased after 6-Gingerol treatment (Figures 3B,C, *p* = 0.003, *p* = 0.017, respectively). It has been reported that microglia cells play a major role in neuroinflammation^[32]^. To identify the effects of 6-Gingerol on neuroinflammation and microglia activation, IL-1β and Iba-1 staining, and TNF-α and Iba-1 staining were performed at 3-day post-HIBI. As expected, we found that the number of IL-1β^+^Iba-1^+^ cells and TNF-α^+^Iba-1^+^ cells in the ipsilateral cortex was increased in HIBI group as compared with the Sham group (*p* < 0.0001), while 6-Gingerol reduced the number of double-positive cells in the ipsilateral cortex (*p* = 0.007, *p* < 0.0001, respectively) (Figures 3D,E). Therefore, these results indicate that 6-Gingerol can attenuate HIBI-induced inflammation.

### 6-Gingerol Improves HIBI-Induced Brain Atrophy and Dyskinesia

**FIGURE 4:**
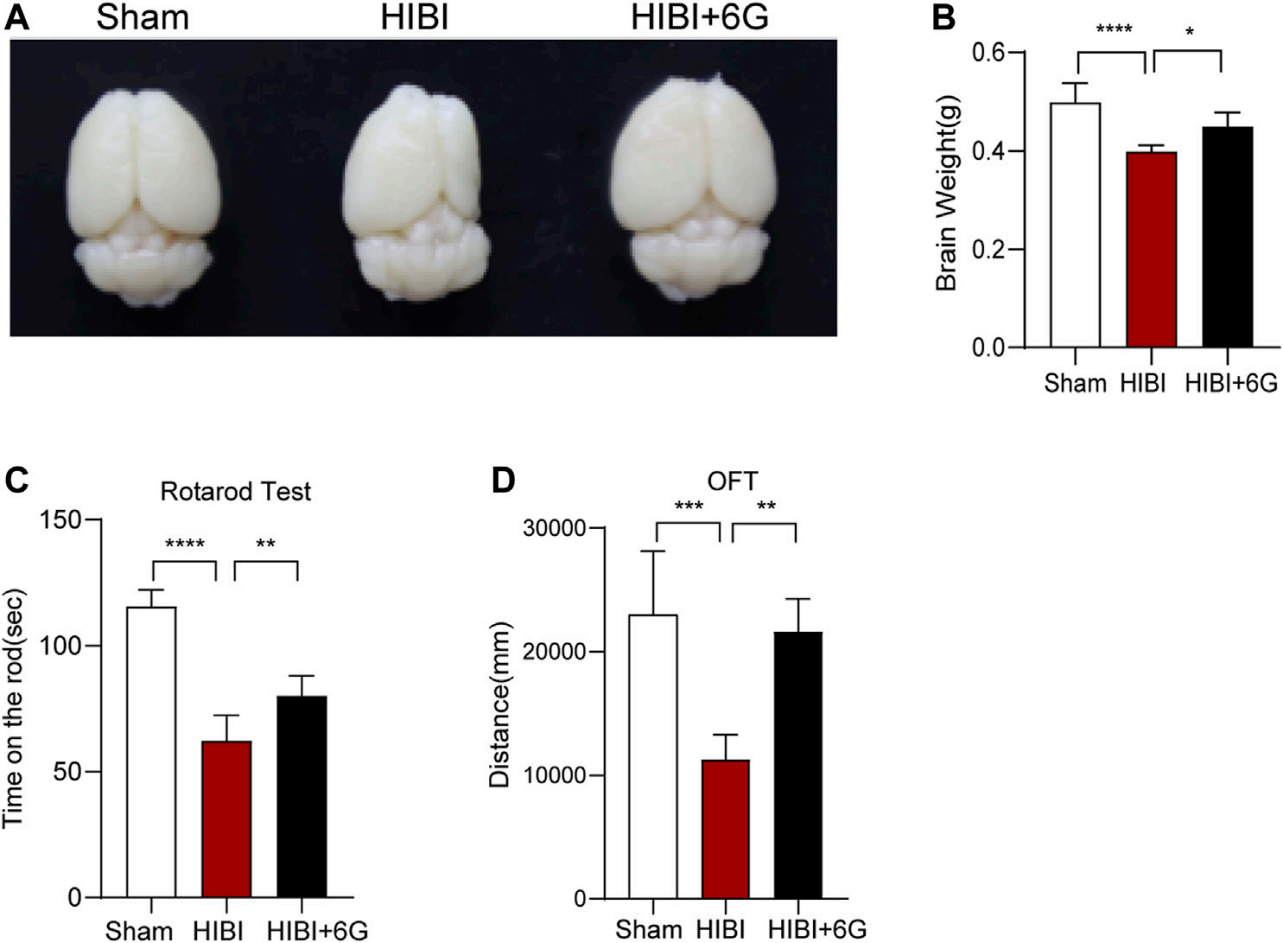
6-Gingerol improves HIBI-induced brain atrophy and long-term neurological deficits. **(A)** Representative images of the anatomical structure of the brains of each group at 4-week post-HIBI. **(B)** Brain weight of each group. **(C)** Rotarod test for the motor coordination evaluation of each group at 4-week post-HIBI. *n* = 6 per group. **(D)** Open field test for total distance results of each group. *n* = 5 per group. Data represent the mean ± SEM. *p < 0.05, **p < 0.01, ***p < 0.001, ****p < 0.0001.

To investigate the beneficial impacts of 6-Gingerol on long-term brain injury post-HIBI, brain weight and atrophy were examined at 4-week post-HIBI. As shown in Figures 4A,B, the HIBI group showed severe atrophy of the lesioned hemisphere and marked brain-weight loss as compared with the Sham group (*p* < 0.0001). Mice treated with 6-Gingerol exhibited less brain-weight loss and brain atrophy (*p* = 0.023). Furthermore, the rotarod test and OFT were used to identify the effects of 6-Gingerol treatment on long-term neurological deficits at 4-week post-HIBI. As shown in Figure 4C, the HIBI group spent less time on the rod than the Sham group (*p* < 0.0001). In contrast, rotation time in the HIBI+6G group was markedly improved (*p* = 0.006). On the OFT, mice in the HIBI+6G group had a longer moving distance than the HIBI group (*p* = 0.002) (Figure 4D). These results suggested that the motor deficit was ameliorated by 6-Gingerol post-HIBI.

### 6-Gingerol Inhibits HIBI-Induced Loss of Myelin

**FIGURE 5:**
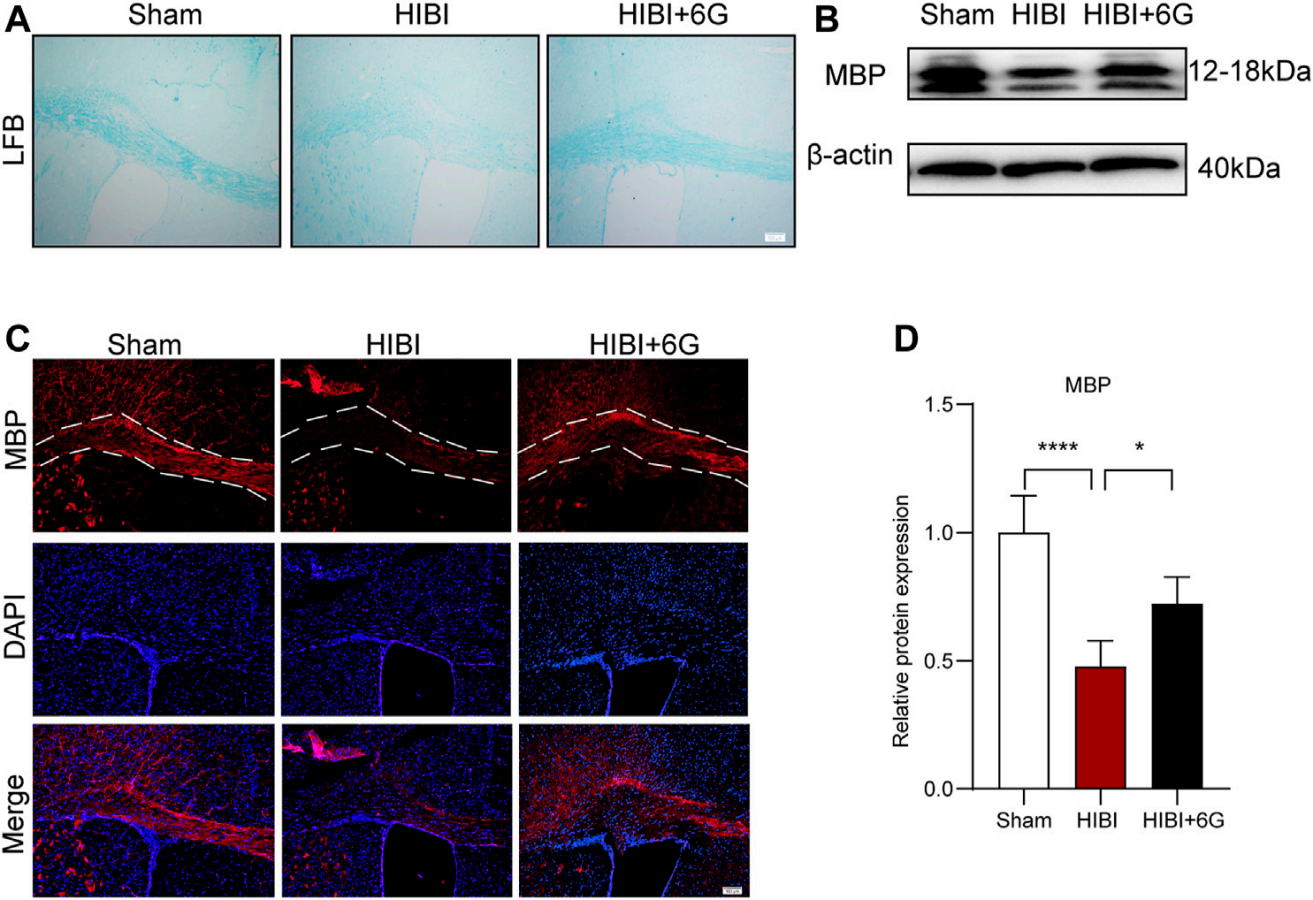
6-Gingerol inhibits HIBI-induced loss of myelin at 4-week post-HIBI. **(A)** Representative Luxol Fast Blue staining of the callosum at 4-week post-HIBI. **(B)** Representative myelin basic protein (MBP) staining of the callosum at 4-week post-HIBI. **(C–D)** Representative western blot images and quantification of MBP expression of the callosum at 4-weeks post-HIBI. *n* = 5 per group. The area marked with dashed lines represents the corpus callosum. Scale bar = 100 μm. Data represent the mean ± SEM. *p < 0.05, **p < 0.01, ***p < 0.001, ****p < 0.0001.

Myelin development is closely related to motor function (Bacmeister et al., 2020). Consequently, we assessed whether 6-Gingerol alleviated the loss of myelin post-HIBI. LFB staining and MBP staining were used to examine myelin lesions in the callosum at 4-weeks post-HIBI (P35). As noted in Figure 5A, slices collected from the HIBI group showed a considerable extent of demyelination, as revealed by the loss of LFB-staining myelin. 6-Gingerol treatment significantly increased the density of LFB-staining myelin. In Figure 5B, mice with HIBI exhibited lower immunofluorescence intensity of MBP-staining, while 6-Gingerol treatment could prevent the decrease of MBP-staining myelin in the ipsilateral callosum post-HIBI. Similarly, the protein level of MBP was analyzed by western blot and the results showed that the expression of MBP was decreased in the ipsilateral callosum post-HIBI (*p* < 0.0001). 6-Gingerol treatment significantly rescued HIBI-induced decrease in MBP (*p* = 0.017). Taken together, our data suggested that 6-Gingerol could prevent the loss of myelin post-HIBI.

### 6-Gingerol Stimulates Oligodendrocyte Maturation and Alleviates Oligodendrocyte Apoptosis

**FIGURE 6:**
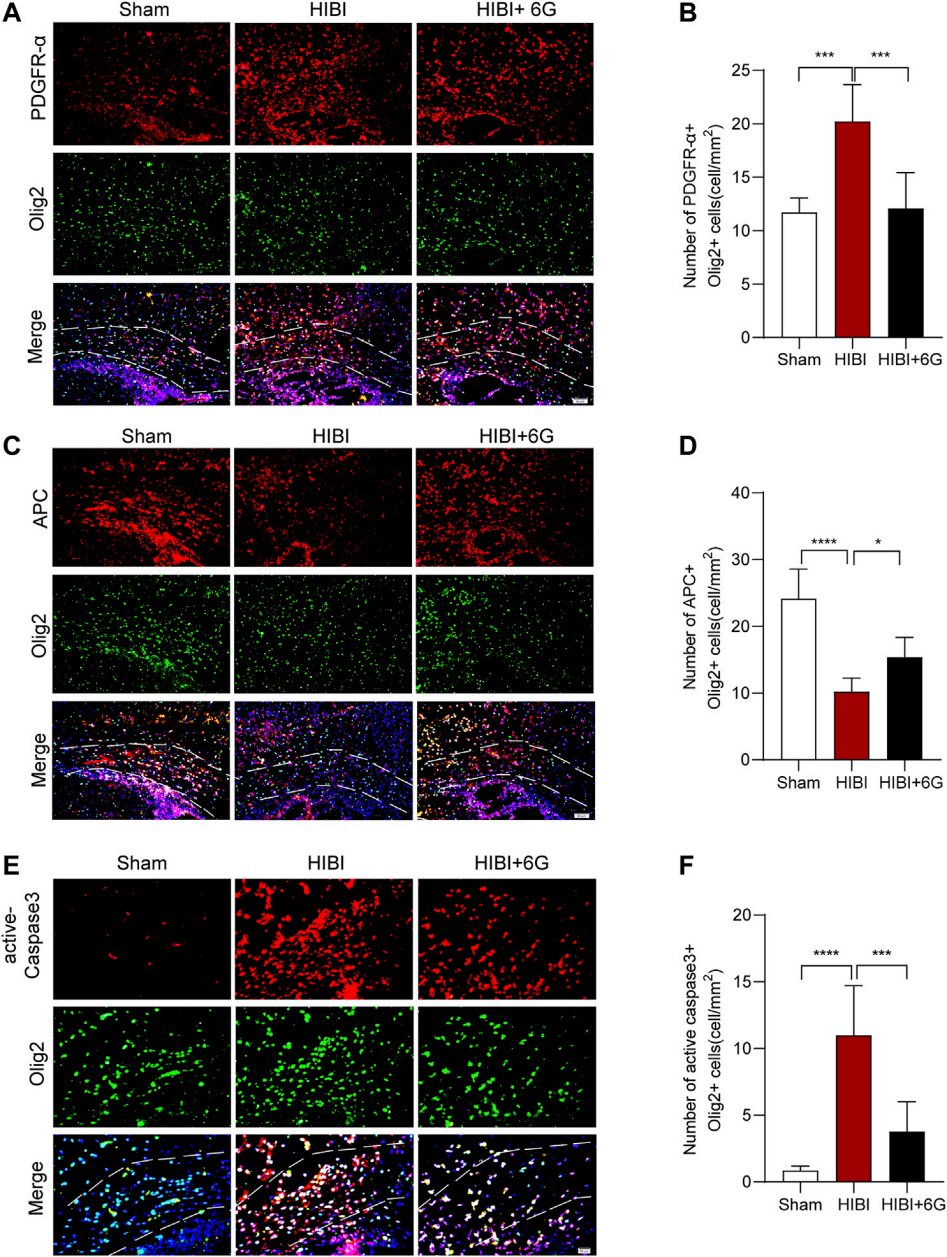
6-Gingerol stimulates the maturation of oligodendrocytes and reduces the apoptosis of oligodendrocytes. **(A)** Representative PDGFR-α stained (red) and Olig2 stained (green) brain sections of the callosum at 3-day post-HIBI. Scale bar = 50 μm. **(B)** Quantitative analysis of the percentage of PDGFR-α^+^Olig2^+^ cells in the callosum. **(C)** Representative APC stained (red) and Olig2 stained (green) brain sections of the callosum at 3-day post-HIBI. Scale bar = 50 μm. **(D)** Quantitative analysis of the percentage of APC+Olig2^+^ cells in the callosum. **(E)** Representative active-Caspase3 stained (red) and Olig2 stained (green) brain sections of the callosum at 3-day post-HIBI. Scale bar = 20 μm. **(F)** Quantitative analysis of the percentage of active-Caspase3+Olig2^+^ cells in the callosum. *n* = 6 per group. The area marked with dashed lines represents the corpus callosum. Data represent the mean ± SEM. *p < 0.05, **p < 0.01, ***p < 0.001, ****p < 0.0001.

Numerous studies have shown that HIBI results in a white matter injury called periventricular leukomalacia, which is the major cause of cerebral and long-term neurological morbidity (Deng, 2010; Jiang et al., 2016). To test the influence of 6-Gingerol treatment on oligodendrocytes, immunostaining of the oligodendrocyte marker (Olig2) and oligodendrocyte precursor cell marker (PDGFR-α) was performed at 3-day post-HIBI (P10). As shown in Figures 6A,B, HIBI significantly increased the number of PDGFR-α^+^Olig2^+^ cells in the callosum (*p* < 0.001), while 6-Gingerol treatment reduced the number of double-positive cells as compared with the HIBI group (*p* = 0.001). This result indicated that HIBI prevented oligodendrocytes from maturing, keeping them in an immature state. Furthermore, dual staining for Olig2 and APC (a marker of mature oligodendrocytes) was performed to evaluate oligodendrocyte maturation post-HIBI. Figures 6C,D showed that HIBI decreased the number of APC-positive (APC^+^)/Olig2^+^ cells (*p* < 0.0001). In the HIBI+6-Gingerol group, more APC^+^/Olig2^+^ cells were observed in the ipsilateral callosum post-HIBI than in the HIBI group (*p* = 0.040). The above results suggested that 6-Gingerol exerted beneficial effects on the maturation of oligodendrocytes by decreasing the number of immature oligodendrocytes and increasing the number of mature oligodendrocytes.

In order to test the influence of 6-Gingerol treatment on oligodendrocyte cell death, Olig2 and active-Caspase3 immunostaining was performed at 3-day post-HIBI. As shown in Figures 6E,F, HIBI significantly increased the number of dual-positive cells in the callosum (*p* < 0.0001), while 6-Gingerol treatment reduced the cell death of oligodendrocytes as compared with the HIBI group (*p* < 0.001).

### 6-Gingerol Exerts Neuroprotective Effects by Regulating AKT, ERK, and NF-κB Pathway, and Epigenetic Modification

**FIGURE 7:**
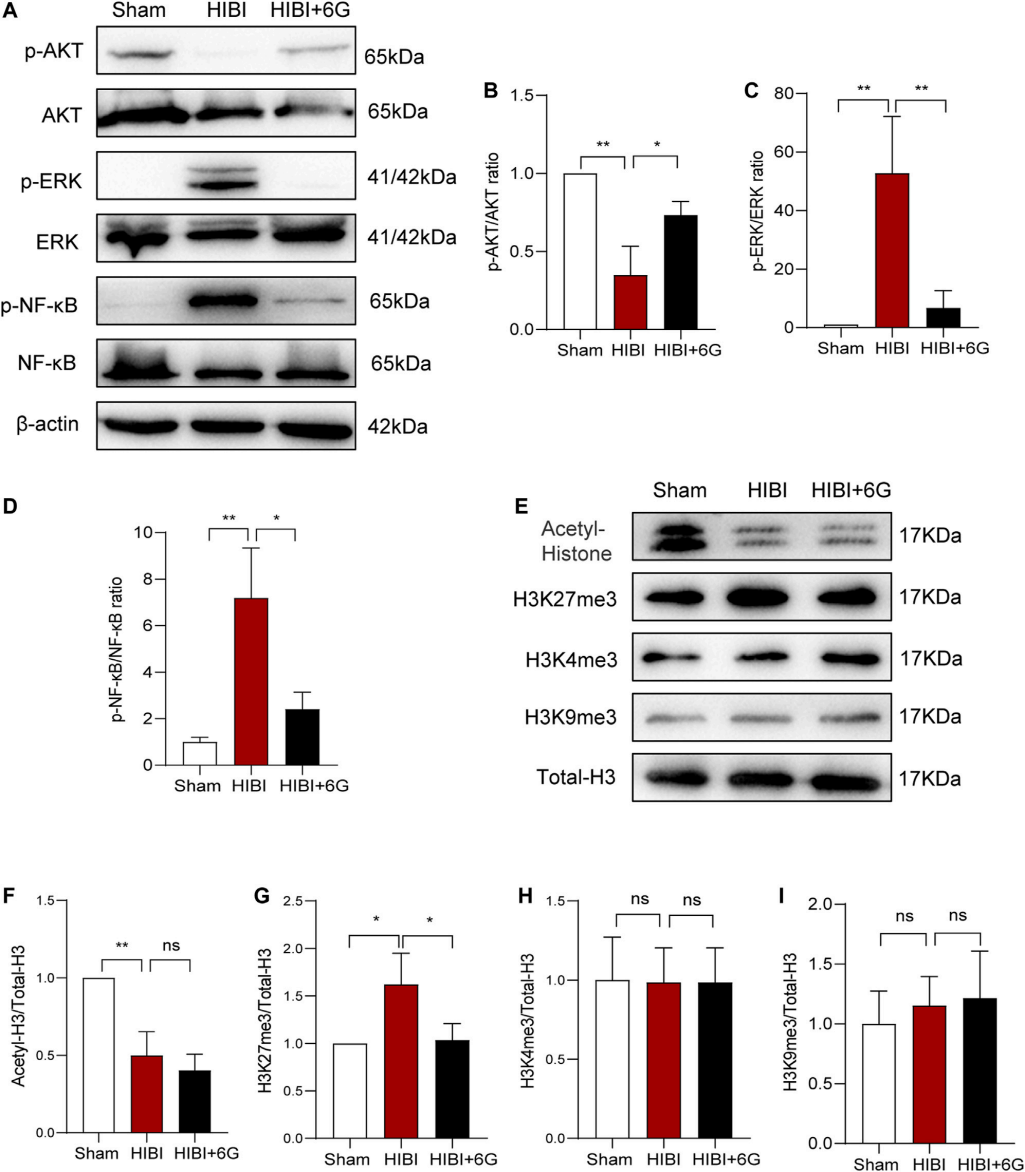
Protein levels related to the AKT, ERK, and NF-κB pathways, and histone acetylation and methylation. **(A–D)** Representative western blot images and quantification of *p*-AKT, *p*-ERK, p-NF-κB expression in the brain. **(E–L)** Representative western blot images and quantification of histone acetylation and methylation expression in the brain. *n* = 3 per group. Data represent the mean ± SEM. *p < 0.05, **p < 0.01, ***p < 0.001, ****p < 0.0001.

Neuroinflammation plays a vital role in hypoxic-ischemic brain injury. Thus, we detected the expression of inflammation-related pathway proteins in the brain at 3-day post-HIBI (Figures 7A–D). Our results showed that the expression level of phosphor-AKT (*p*-AKT) was significantly downregulated in comparison with that of the Sham group (*p* = 0.001), while 6-Gingerol activated the AKT pathway and improved the expression of *p*-AKT levels (*p* = 0.017). In addition, the ERK pathway was activated after HIBI (*p* = 0.004) and 6-Gingerol could reverse this change (*p* = 0.007). Moreover, the level of phosphor-NF-κB (p-NF-κB) was increased post-HIBI (*p* = 0.003). However, 6-Gingerol could affect activation of the NF-κB pathway (*p* = 0.010). These data substantiated that 6-Gingerol could exert a protective effect through regulation of AKT, ERK, and NF-κB signaling.

Epigenetic modification plays a crucial role in gene expression regulation and participates in a variety of physiological and pathological processes (Creyghton et al., 2010; Moon et al., 2020). Therefore, we examined changes in histone acetylation and methylation at 3-day post-HIBI (P10). As shown in Figure 7E, the expression level of total histone acetylation was significantly decreased after HIBI (*p* = 0.003). However, 6-Gingerol treatment did not alter the histone acetylation level induced by HIBI. We also detected histone methylation at important sites such as, H3K4me3, H3K9me3, and H3K27me3. The results showed that the level of H3K27me3 in the HIBI group was significantly increased (*p* = 0.029), but the changes in H3K4me3 and H3K9me3 were not significant. 6-Gingerol treatment could markedly inhibit HIBI-induced increase in H3K27me3 (*p* = 0.036). Taken together, these results showed that 6-Gingerol could play a neuroprotective role by regulating epigenetic modification.

## Discussion

Brain damage induced by hypoxic-ischemic injury during fetal or neonatal periods is a major factor in neonatal mortality and in neurological deficits of the newborn. At present, HIE is mostly treated with hypothermia, but the effect is not ideal. Many sick children develop permanent nervous system sequelae, such as motor disorders, intellectual disability, and so on. Therefore, there is an urgent need to explore new treatments. Recent studies have shown that 6-Gingerol, as a new Chinese herbal medicine in nervous system therapy, has significant therapeutic anti-inflammatory, anti-apoptotic and anti-oxidative stress effects (Ren et al., 2019; Sun et al., 2020; Zahoor et al., 2020). In present study, we explored the therapeutic effect of 6-Gingerol in HIBI model, defined its benefical effects in acute and long-term injury. In the acute phase, 6-Gingerol could attenuate neonatal HIBI and improve short-term reflex performance by decreasing cell apoptosis and inhibiting inflammatory responses. In the long-term effect, we found that 6-Gingerol could significantly improve motor ability and reduce myelin loss by alleviating oligodendrocyte apoptosis and stimulating their maturation. In terms of the underlying mechanism, we demonstrated that 6-Gingerol could accurately regulate inflammatory pathways and the level of histone H3K27me3, so as to achieve a variety of beneficial effects.

A noteworthy feature in the present results was the protective effect of 6-Gingerol on brain structural damage caused by HIBI and the contribution to functional recovery. Consistent with previous studies, the application of 6-Gingerol played an important neuroprotective role in ischemic brain injury. However, previous study has focused on the role of 6-Gingerol in adult ischemic stroke (Liu et al., 2020). The present study provides a basis for the application of 6-Gingerol in neonatal nervous system diseases through neonatal HIBI model. Together with previous study, it is proved that 6-Gingerol plays an important role both in adult stroke and neonatal HIBI. In the early development, 6-Gingerol treatment improved the short-term brain damage and neurological reflex performance in mice. The brain experiences acute inflammatory response in the early stage of HIBI. Previous studies have shown that 6-Gingerol can reduce neuroinflammatory responses in primary peripheral nerve injury in mice and play an anti-inflammatory role in mastitis and myocardial fibrosis (Borgonetti et al., 2020; Han et al., 2020; Zahoor et al., 2020). In hypoxia-induced cardiomyocyte injury, 6-Gingerol can enhance the vitality of cardiomyocytes, and reduce apoptosis and oxidant stress, thereby protecting cardiomyocytes from hypoxic damage (Ren et al., 2019). The above studies have fully suggested that 6-Gingerol can play a protective role in hypoxia induced cell injury and neuroinflammation response. Consistent with these previous studies, we found that HIBI-induced cell apoptosis could be rescued by 6-Gingerol. Moreover, in the early inflammatory response, the administration of 6-Gingerol reduced the release of pro-inflammatory factors post-HIBI. Microglia cells are the main producer of inflammation in HIBI (Li et al., 2017). We also found that 6-Gingerol can reduce the activation of microglia in the early stage of injury. However, glial cells in the nervous system are rich in types and diverse in functions. In addition to microglial inflammation and neuronal damage, other glial cell types need to be deeply studied.

Furthermore, we found that 6-Gingerol could also improve long-term brain atrophy and motor dysfunction in mice post-HIBI, which further proves the effective role of 6-Gingerol in HIBI. Surviving children with HIBI are usually accompanied by serious neurological sequelae, which affect the life quality of infants and bring a heavy burden to the society and family (Eunson, 2015). This improvement of 6-Gingerol makes it have potential clinical application value. In this study, we found that 6-Gingerol could improve myelin damage, increase MBP expression, and reduce demyelination in the late stage of injury after HIBI. White matter is involved in motor function, and white matter injury is considered to be one of the main pathological changes of multiple nervous system injuries (Bacmeister et al., 2020). Moreover, with the continuous in-depth study of white matter in complex brain functions such as learning, memory and emotion, reducing white matter injury become a potential target to further promote the functional recovery after nervous system damage (Xin et al., 2020; Go et al., 2021; Luo et al., 2021). Although a previous study has reported that 6-Gingerol can alleviate demyelination induced by multiple sclerosis, the specific mechanism of the oligodendrocytes involved in myelin injury has not been explored (Han et al., 2019). Numerous studies have shown that reducing myelin loss and promoting oligodendrocyte maturation are potential mechanisms for improving demyelination damage (Carloni et al., 2020; Xie et al., 2020). The peak of oligodendrocyte development is about 2–6 weeks after birth. Through the proliferation and differentiation of oligodendrocyte precursor cells, mature oligodendrocytes are formed to play the function of myelination (Back, 2017; Elbaz and Popko, 2019). The onset of neonatal HIBI just covers the stage of oligodendrocyte development. HIBI is also a very good injury model to study the development process of oligodendrocytes. Early and appropriate intervention can greatly reduce the damage to the development of oligodendrocytes, so as to save the myelination process and reduce the occurrence of related sequelae (Altamentova et al., 2020; Kaminski et al., 2020). We here showed that, dating back to the early stage of Hibi injury, 6-Gingerol could promote the maturation of oligodendrocytes by promoting the differentiation of oligodendrocytes, which provides great help for the recovery of nervous system function in the later stage.

Finally, we found that 6-Gingerol played a neuroprotective role in HIBI by regulation of the AKT, ERK, and NF-κB pathways and of epigenetic modification. Recently, studies showed that the protective effect of 6-Gingerol was related to important inflammation-related pathways, including NF-κB, MAPK and AKT (Han et al., 2020; Xu et al., 2020) pathways. Inflammatory reaction continues after HIBI producing secondary brain injury (Liu and McCullough, 2013). To explore the potential mechanisms of 6-Gingerol in HIBI, we tested these important inflammation-related pathway molecules. Application of 6-Gingerol significantly increased the level of AKT phosphorylation, and inhibited HIBI-induced activation of the ERK and NF-κB pathway, which was consistent with previous studies. In this study, the phenotype of HIBI mice has been improved in many aspects after the application of 6-Gingerol. These changes in cell fate and gene expression can not be explained by only one mechanism or several signal pathways. In the early stage of injury, it involves the dynamic and extensive regulation of a variety of biological processes (Douglas-Escobar and Weiss, 2015). More and more evidences showed that epigenetic-modification regulates gene activity without changing gene structure, which is closely related to a number of biological processes (Creyghton et al., 2010; Moon et al., 2020). In the present study, we found for the first time that 6-Gingerol played a neuroprotective role in HIBI by epigenetic modification. In epigenetic research, histone modification is an important component of epigenetic regulation, and has become a focus of research into the occurrence of neuroinflammation, nerve cell apoptosis and nervous system diseases (Lee et al., 2020; Jiang et al., 2021). Therefore, we tested whether 6-Gingerol exerted neuroprotective effects through epigenetic modification, and analyzed the changes of the relevant histones. We found that 6-Gingerol did not affect the acetylation of histones, but regulated the methylation level of H3K27me3. Similarly, a previous study has shown that the expression of H3K27me3 was downregulated at 24-h post-HIBI, while sevoflurane post-conditioning significantly increased the expression of H3K27me3, alleviating cerebral injury (Xue et al., 2019). In our study, we found that the expression level of H3K27me3 was increased at 72-h post-HIBI, suggesting that H3K27me3 may dynamically participate in the different processes of HIBI to exert neuroprotective effects. Epigenetic regulation has a wide range of effects and can regulate multiple genes expression (Creyghton et al., 2010; Moon et al., 2020). H3K27me3 modification may be involved in microglia activation, oligodendrocyte development and neuroprotection and other processes, and the specific regulatory targets are worthy of further study. Importantly, the change pattern of H3K27me3 in the dynamic process of HIBI is clarified through the treatment of 6-Gingerol, which may provide a broader therapeutic target for the treatment of such diseases.

In conclusion, our work revealed that 6-Gingerol reduces short-term and long-term brain injury and restores neurobehavioral function after HIBI in neonatal mice by reducing cell death, restraining microglia activation, and improving axonal hypomyelination. Further analysis revealed that 6-Gingerol regulated AKT, ERK, and NF-κB pathways and epigenetic modification. Therefore, our exploration of the role of 6-Gingerol in HIBI has provided some insights into the clinical treatment of neonatal HIE.

## Data Availability

The original contributions presented in the study are included in the article/Supplementary Material, further inquiries can be directed to the corresponding author.

## Abbreviations

HIBI, hypoxic-ischemic brain injury; HE, Hematoxylin and eosin; LFB Luxol Fast Blue; MBP, Myelin basic protein; OFT, Open-field test; TTC, Triphenyl tetrazolium chloride.
